# Supplementary material for: Efficacy, Safety, and Concerns on Microbiota Modulation, Antibiotics, Probiotics, and Fecal Microbial Transplant for Inflammatory Bowel Disease and Other Gastrointestinal Conditions: Results from an International Survey
Source: Microorganisms. 2023 Nov 19;11(11):2806. doi: 10.3390/microorganisms11112806 (PMC10672996; doi:10.3390/microorganisms11112806)
Supplement: Supplementary file 1 [file microorganisms-11-02806-s001.zip › microorganisms-2684352-supplementary.pdf]

Project: Fecal microbial transplant and probiotics in the treatment of inflammatory bowel diseases - IBD Physicians survey

Demographic of respondents

1) Year of birth

2) Gender
   a) Man
   b) Woman
   c) Other
   d) I prefer not to disclose

3) In which country do you practice?

4) What is your specialty?
   a) Trainee
   b) Adult gastroenterologist specialist
   c) Pediatric gastroenterologist specialist
   d) Surgeon
   e) Other

5) Where do you work?
   a) University Hospital
   b) Tertiary non-academic hospital
   c) Secondary hospital
   d) Private practice
   e) Other

6) Years of experience in the field of IBD
   a) Less than 1 year
   b) Between 1 and 5 years
   c) Between 5 and 10 years
   d) More than 10 years

7) Number of IBD patients visited per year
   a) Less than 100
   b) Between 100 and 500
   c) Between 500 and 1000
   d) More than 1000

Knowledge of microbiota and its relevance to IBD

8) What is your overall knowledge of microbiota
a) Very limited
b) Limited
c) Medium
d) Quite knowledgeable
e) Expert

9) How relevant do you think microbiota is to the pathogenesis of IBD? (0 not relevant at all; 10 extremely relevant)

10) Do you use microbiota composition analysis in your practice?
a) Regularly
b) Often
c) Occasionally
d) Never

11) If you don't use microbiota analyses, why? Multiple options possible
a) No application to clinical practice
b) Not reimbursed by insurance/health care system
c) I wouldn't know how to interpret the results
d) Lack of reference values and high variability (intrapatient and interpatients)
e) Other

Microbiota modulation

12) For which indications do you prescribe probiotics? (0 never, 10 always)
a) Ulcerative colitis
b) Crohn's disease
c) Pouchitis
d) After a gastrointestinal infection
e) Parallel to an antibiotic course for a non-gastrointestinal indication
f) Irritable bowel syndrome with constipation
g) Irritable bowel syndrome with diarrhea

13) Do you think antibiotics are useful/effective in inducing remission of IBD (excluding fistulas and abscess)?

14) Do you think antibiotics are useful/effective in maintaining remission of IBD (excluding fistulas and abscess)?

15) Do you think that certain antibiotics cause flares of IBD?

Fecal microbiota transplantation (FMT) in IBD

17) How effective do you think FMT could be for the following conditions (0 never, 10 always)
   a) Ulcerative colitis
   b) Crohn’s disease
   c) Pouchitis
   d) Irritable bowel syndrome with constipation
   e) Irritable bowel syndrome with diarrhea
   f) Other

18) How efficacious do you consider FMT compared to biologics/advanced small molecules?
   a) More effective than biologics/advanced small molecules
   b) As effective as advanced therapies
   c) Less effective than advanced therapies
   d) I don't know

19) How often do you think FMT should be repeated in patients with IBD to maintain remission?
   a) Once
   b) Weekly
   c) Monthly
   d) Every 2 months
   e) Every 6 months
   f) Yearly
   g) Not sure

20) Do you think that the FMT donor is relevant for the therapeutic success (“super donor concept”)
   a) Yes
   b) No
   c) Not sure

21) Where would you place FMT in the treatment algorithm of IBD?
   a) First line treatment
   b) Second line (after a first line medication failed)
   c) After medical treatment and before surgery
   d) Don’t know

22) If it was available and reimbursed would you consider using FMT in patients with IBD?
   a) Yes
   b) Only for very selected patients
   c) Not yet, I want to see more data
   d) No

e) Not sure

23) Would consider enrolling patients into clinical trials evaluating FMT for IBD?
   a) Yes
   b) No
   c) Not sure

24) Do you think FMT is safe?
   a) Yes
   b) No
   c) Undecided

25) What are your concerns about the use of FMT in IBD patients or why you would not refer a patient? (multiple answers possible)
   a) Sepsis/infection risk
   b) Risk of transmission of disease undetected by screening procedures
   c) Acceptability of treatment by patient
   d) Lack of evidence of efficacy
   e) Lacking clinical evidence to support its safety
   f) Absence of guidelines
   g) Administration and unpleasant nature of FMT
   h) Ethics
   i) Other

27) What are the most important factors to consider when deciding whether to recommend FMT as a treatment for IBD?
   a) Type of IBD (UC vs CD)
   b) Response to previous IBD treatment
   c) Disease severity
   d) Infectious risk
   e) Availability of other medical treatments

Personal experience (targeted at direct experience)

28) Have you previously performed FMT?
   a) Yes, personally
   b) No, but my center offer/perform FMT
   c) No, FMT is not performed in my center
   d) I have never heard of FMT before this survey

IF NO → END OF SURVEY

29) If yes, for which indication? (multiple answers possible)

a) Crohn's disease
b) Ulcerative colitis
c) Irritable bowel syndrome
d) Others

30) If yes, did you find FMT effective?
a) Yes, very effective
b) Yes, somewhat effective
c) Neither beneficial nor detrimental
d) Detrimental (it worsen the conditions)
e) Not sure

31) If yes, did you find FMT easy to administer/organize?
a) Yes
b) No

32) If yes, which administration route did you use? Multiple options possible
a) Oral tablets/capsules/Enteric coated capsules
b) Nasogastric/duodenal tube
c) Upper GI endoscopy
d) Rectal enema
e) Lower GI endoscopy
f) Other

33) If performing FMT for IBD, are you combining it with other therapeutic interventions?
a) No, alone
b) With the going IBD treatment
c) Probiotics
d) Antibiotics
e) Prebiotics
f) Symbiotics
g) Nutrition

34) In which disease phase you think FMT should be used?
a) To induce remission
b) To maintain remission
c) Both

35) What aspect(s) of FMT are you less comfortable with? Several answers possible
a) Indications (unclear evidence)
b) Stool preparation
c) Stool administration
d) Safety
e) Talking to patients about it

Thank you for your participation
